# Monitoring therapeutic response to tamoxifen in NMU-induced rat mammary tumours by 31P MRS.

**DOI:** 10.1038/bjc.1991.198

**Published:** 1991-06

**Authors:** S. Baluch, C. J. Midwood, J. R. Griffiths, M. Stubbs, R. C. Coombes

**Affiliations:** CRC Biomedical Magnetic Resonance Research Group, St. George's Hospital Medical School, London, UK.

## Abstract

Tamoxifen injections were given once a week for 4 weeks to 19 rats bearing N-methyl-N-nitrosourea (NMU)-induced mammary carcinomas. NMR spectra were collected on days 2, 7, 14, 21 and 28. Only 42% of the tumours responded to the tamoxifen in that they regressed significantly; another 21% did not change in size and 37% grew significantly. In the ones that did subsequently regress there were significant changes in the NTP/Pi ratio as early as 2 days after treatment, before any detectable change in volume was recorded, and continuing up to 21 days. The significance of these findings and the possible mechanisms underlying the changes are discussed.


Br. J. Cancer (1991), 63, 901-904 © Macmillan Press Ltd., 1991

### Monitoring therapeutic response to tamoxifen in NMU-induced rat mammary tumours by $^{31}$P MRS

S. Baluch[1], C.J. Midwood[1,†], J.R. Griffiths[1], M. Stubbs[1] & R.C. Coombes[2,*]

[1]*CRC Biomedical Magnetic Resonance Research Group, and* [2]*Clinical Oncology Unit, St. George's Hospital Medical School, Cranmer Terrace, London SW17 0RE, UK.*

**Summary** Tamoxifen injections were given once a week for 4 weeks to 19 rats bearing N-methyl-N-nitrosourea (NMU)-induced mammary carcinomas. NMR spectra were collected on days 2, 7, 14, 21 and 28. Only 42% of the tumours responded to the tamoxifen in that they regressed significantly; another 21% did not change in size and 37% grew significantly. In the ones that did subsequently regress there were significant changes in the NTP/Pi ratio as early as 2 days after treatment, before any detectable change in volume was recorded, and continuing up to 21 days. The significance of these findings and the possible mechanisms underlying the changes are discussed.

Endocrine treatments are widely used in advanced breast cancer, but only cause remission in about 30% of patients. Some indication as to the likelihood of response is given by the measurement of tumour hormone receptor status (e.g. oestrogen receptor (ER) or progesterone receptor (PR)) but about 50% of ER-positive patients fail to respond, and about 5% of ER-negative patients do respond. Furthermore, the median duration of response is only 20 months (Powles, 1984).

There is clearly a need for a rapid and sensitive indicator of tumour response. Unfortunately decrease in tumour size, as a measure of responsiveness to endocrine therapy, tends to be slow; Powles (1984) quotes times ranging from 15 weeks in soft tissues to 41 weeks in bone to achieve a partial response. The technique of Magnetic Resonance Spectroscopy (MRS) offers a way to measure the response of tumours to various therapeutic modalities (for review see Steen, 1989). In studies performed on oestrogen-sensitive rat mammary tumours (Rodrigues *et al.*, 1988) we have shown that ovariectomy induced a marked change in phosphate metabolite ratios (PCr/NTP, NTP/Pi, PCr/Pi) that could be detected at 48 h, well before any measurable regression or significant histological changes (Stubbs *et al.*, 1990) had taken place. This raised the possibility that MRS (which is non-invasive and has no known harmful effects on patients) could be used to monitor response to endocrine therapy. Tumours that failed to respond could be treated immediately by another modality, instead of allowing several weeks or months for growth and dissemination to take place.

In current medical practice the most commonly used agent in endocrine therapy of breast cancer is tamoxifen, an oestrogen antagonist. In the present paper we report studies on the use of MRS to monitor the response of NMU-induced oestrogen-sensitive rat mammary tumours to tamoxifen.

### Materials and methods

### *Animals and tumours*

Oestrogen sensitive mammary tumours were induced in female virgin Ludwig/Wistar/Olac rats with N-methyl-N-nitrosourea (NMU) as detailed in Stubbs *et al.* (1990). After about 12 weeks 80% of the animals developed mammary tumours. The tumour volumes were measured using the following formula:

$$v = \pi/6\ (d1.d2.d3)$$

where d1, d2 and d3 are the length, width and depth of the tumour.

### *Tamoxifen*

Tamoxifen (Nolvadex) was obtained from ICI PLC, Pharmaceuticals Division, Unit 6, Cumberland Avenue Estate, NW10. Tablets, which are not soluble in water, were homogenised in Mazola oil and doses of 0.01 mg $kg^{-1}$ were given intramuscularly each week for 4 weeks.

### *NMR measurements*

Tumours were examined by NMR spectroscopy when their size was more than 1.5 $cm^3$ (range 1.66–2.64 $cm^3$). The animals were anaesthetised with pentobarbitone (30 mg $kg^{-1}$ i.p.) and placed within the 27 cm bore of 1.89 Tesla Oxford Research System-TMR-32 200 instrument. Spectra were obtained at 32 MHz using a 1 or 1.4 cm diameter surface coil with a pulse width of 6 or 8 μs respectively, a 3 s repetition time and 480 scans. Peak areas were calculated using the software package supplied with the instrument after profile correction to remove some of the broad signal. Due to difficulties in baseline definition and overlapping peaks these integrals may not give true chemical concentrations and for this reason the results are expressed as ratios of integrals which minimises some of the uncertainties. The reproducibility of the integrations was $100 \pm 0.3\%$ (10) (mean ± s.e.m.). PCr was not consistently present in this set of tumours and therefore the results reported are limited to the integral of the βNTP peak (which includes contributions mainly from ATP and GTP – see Stubbs *et al.*, 1989) relative to the Pi integral i.e. βNTP/Pi with means ± s.e.m.

### Results

A total of 39 animals were used in this study. Twenty-five were treated with tamoxifen and 14 were used as controls; six of the animals died during the experiments due to the sensitivity of these animals to anaesthesia, and were not included in the results. The tumours were monitored both for volume changes (see Figure 1) and by $^{31}$P spectroscopy (see Figures 2 and 3) at 2, 7, 14, 21 and 28 days. Of the treated animals, eight responded showing regression to less than 50% of their original starting volume (see Figure 1), four animals showed no further growth, whilst seven did not respond to the

Present addresses: *Department of Oncology, Charing Cross Hospital, St. Dunstan's Road, London W6 8RP; †Royal Marsden Hospital, Fulham Road, London SW3 6JJ, UK.
Correspondence: M. Stubbs, CRC Biomedical Magnetic Resonance Research Group, St. George's Hospital Medical School, Cranmer Terrace, London SW17 0RE, UK.
Received 27 November 1990; and in revised form 24 January 1991.

**Figure 1** The effect of tamoxifen on the volumes of rat mammary tumours. The volumes prior to treatment were taken to the 100% and the subsequent changes were expressed (mean ± s.e.m.) relative to the measurements made at day 0. ●——● responders ($n$ = 8), □——□ non responders ($n$ = 7) and ○——○ ($n$ = 14) controls. *$P$ <0.01 compared to controls.

**Figure 3** The effect of tamoxifen on NTP/Pi ratios in rat mammary tumours. The volumes prior to treatment were taken to be 100% and the subsequent changes were expressed (mean ± s.e.m.) relative to the measurements made at day 0. ●——● responders, □——□ non responders and ○——○ controls. *$P$ <0.05 compared to controls.

**Figure 2** The effect of tamoxifen on $^{31}$P NMR spectra of rat mammary tumours. Assignments as follows: 1. Phosphomonoesters. 2. Pi. 3. Phosphodiesters. 4. γ NTP. 5. α NTP. 6. β NTP. The spectra are representative of the responding group.

treatment and continued to grow, as did all the controls. The volumes of the responders were significantly different from the controls ($P$ >0.01) at all time points except Day 2. Volume measurements showed that the non-responding tumours grew at a similar rate to the controls (see also Figure 1).

Spectra acquired on days 0, 7 and 14, and representative of the responding group, are shown in Figure 2. βNTP/Pi ratios were measured from the spectra; it is apparent from examination of the spectra that the change in the ratio occurred because Pi decreased, and not because NTP increased (see Figure 2). The results of all the experiments (shown in Figure 3) show that these changes were significant when compared either to the controls or to the non-responders at 2, 7, 14 and 21 days ($P$ <0.05) although not significant at 28 days.

The tumours in the treated group that did not regress, and remained similar in size to the starting volume (only 4), showed a significant increase in the NTP/Pi ratios at 2 days, but were not significant at any other time point when compared to the controls (not shown in Figure 3).

### Discussion

In several studies on growing and regressing animal tumours it has been shown that the high energy phosphate content relative to Pi decreases as the tumours increase in size (Evanochko et al., 1984; Rofstad et al., 1988). We and others have attributed this to reduced oxygen delivery, due to the tumours outgrowing their blood supply (Griffiths et al., 1987). This hypothesis is consistent with other results on the metabolism of experimental tumours. For instance, Vaupel et al. (1987) found that small human mammary carcinoma xenografts grown in nude rats have a higher oxygen consumption than that of normal post-menopausal human breast and that both oxygen consumption and tumour blood flow decreased as the tumours increased in size. On the other hand, when a tumour responds to chemotherapy (Ng *et al.*, 1982; Steen *et al.*, 1988), radiotherapy (Evanochko *et al.*, 1983; Tozer *et al.*, 1989) or endocrine therapy (Griffiths *et al.*, 1987; Rodrigues *et al.*, 1988; Stubbs *et al.*, 1990) the opposite effect is usually observed – the ratios of high energy phosphates (PCr and NTP) relative to Pi decrease, sometimes before any detectable change in the volume is recorded (Rodrigues *et al.*, 1988) and before there are any detectable histological changes (Stubbs *et al.*, 1990).

Steen (1989) has reviewed reports of such paradoxical improvements in the ratio of high energy phosphates to Pi in experimental tumours after various forms of therapy, which he terms 'metabolic activation'. He lists five hypotheses to account for the effect: (a) killing of tumour cells and recruitment of host macrophages, thus increasing the proportion of normal cells *vs* cancer cells; (b) killing tumour cells, reducing competition for nutrients and oxygen; (c) preferential killing of 'low energy' cells or recruitment of quiescent cells into a metabolically more active form; (d) cell killing reducing tumoural interstitial pressure, thus improving blood flow; (e) chemotherapy directly increasing tumour blood flow or capillary permeability.

We have argued (Griffiths *et al.*, 1987; Rodrigues *et al.*, 1988) that the very rapid (<2 days) increase in NTP/Pi in tumours that repond to endocrine treatment suggests that as the stimulus to growth (oestrogen in this case) ceases, the demand for ATP falls and the supply of oxygen and other nutrients is again sufficient (Griffiths *et al.*, 1987). Alternatively, or in addition to this mechanism, the tumour blood supply might be improved either directly by tamoxifen itself or as a consequence of reduced oestrogen concentration. We have shown, in the case of ovariectomy, that the MRS changes at 48 h precede any alteration in the cell population (Stubbs *et al.*, 1990). It is likely that this would be true for other endocrine treatments such as 4-hydroxyandrostenedione (Griffiths *et al.*, 1987) and tamoxifen (this paper). If so, it would appear that these early changes cannot be accounted for by mechanismss (a), (b) and (d) suggested by Steen. Mechanism (c) includes two hypotheses: preferential killing of low energy cells (excluded) and recruitment of 'quiescent' cells into a metabolically more active form. The latter hypothesis seems to assume that tumours contain a population of cells in which low NTP or PCr, or high Pi, or both are due, not to an inadequate oxygen or nutrient supply, but to a reversible control mechanism. No metabolic control mechanisms of this kind have been described, to our knowledge. Mechanism (e) assumes a direct effect (either of tamoxifen itself, or by reduction in oestrogen concentration in the present case) on tumour vasculature. There is no evidence for this hypothesis in the present instance, but it cannot be excluded. Radiotherapy appears to increase NTP/Pi by improving tumour blood flow (Tozer *et al.*, 1989).

The reduction in ATP demand that we have postulated after endocrine therapy would also be expected to occur after other treatment modalities that suppress tumour growth. It should therefore be included in the list of factors that give rise to the overall change in the $^{31}P$ spectrum of the tumour. In other cases one of the other factors may play a more important role.

Since MRS is a non-invasive technique, determination of tumour metabolic status during anticancer therapy may be clinically useful, provided that these effects can be reproduced in patients. The early rise of NTP/Pi ratios in the tumours which responded to the tamoxifen treatment and the fall in those which did not respond, could assist the choice of the most appropriate treatment for individual patients. In the case of tumours that did not respond the switch in the treatment would be more rapid than at present when one has to wait for measurable tumour regression to suggest the eventual response. Information of this kind would also be useful in testing drug analogues and dose schedules.

Our hypothesis to explain the rise in NTP/Pi observed in rat tumours soon after initiation of endocrine therapy is that the reduction in demand for oxygen and other nutrients following cessation of growth allows repletion of high energy phosphate pools and a reduction in accumulated Pi. Would human tumours be expected to show this effect? It seems clear that the energy demand associated with tumour cell growth would be reduced following successful endocrine therapy. However, this might not be manifest in human breast cancers if their energy requirements during normal growth can be met by their blood supply.

Steen's review (1989) lists a series of therapeutic studies in patients monitored by $^{31}P$ MRS and reports that increases in the NTP/Pi and/or PCr/Pi ratios occurred in the majority of cases. Ng *et al.* (1989) reported three cases of advanced mammary tumours treated with combined chemotherapy and radiotherapy. In all three, the PME/ATP and phosphodiester/ATP ratios decreased after therapy. Glaholm *et al.* (1989) have reported that the major change in the $^{31}P$ MRS spectrum of a human breast tumour after tamoxifen therapy was a fall in the phosphomenoester peak; the αNTP peak decreased in size, though less markedly than the PME peak. These results are too preliminary to provide a definitive answer to the question posed in a previous paragraph. Larger studies will be needed to show whether the effect we see in rat tumours commonly occur in patients.

The work reported in this paper was supported by the Cancer Research Campaign, UK.

**References**

EVANOCHKO, W.T., NG, T.C. & GLICKSON, J.D. (1984). Application of in vivo NMR Spectroscopy to Cancer. *Magn. Resn. Med.*, **1**, 508.

EVANOCHKO, W.T., NG, T.C., LILLY, M.B. & 4 others (1983). *In vivo* $^{31}P$ NMR study of the metabolism of murine mammary 16C adenocarcinoma and its response to chemotherapy, X-radiation and hypertheramia. *Proc. Natl Acad. Sci. USA*, **80**, 334.

GLAHOLM, J., LEACH, M.O., COLLINS, D.J. & 5 others (1989). *In vivo* $^{31}P$ magnetic resonance spectroscopy for monitoring treatment response in breast cancer. *Lancet*, June 10th, **i**, 1326.

GRIFFITHS, J.R., BHUJWALLA, Z., COOMBES, R.C. & 10 others (1987). Monitoring cancer therapy by NMR Spectroscopy. *Ann. NY Acad. Sci.*, **508**, 183.

NG, T.C., EVANOCHKO, W.T., HIRAMOTO, R.N. & 6 others (1982). $^{31}P$ NMR spectroscopy of *in vivo* tumours. *Magn. Reson. Med.*, **49**, 271.

NG, T.C., GRUNDFEST, S., VIJAYAKUMAR, S. & 7 others (1989). Therapeutic response of breast carcinoma monitored by $^{31}P$ MRS *in situ*. *Magn. Reson. Med.*, **10**, 125.

POWLES, T.J. (1984). *Present Role of Hormone Therapy in Breast Cancer: Diagnosis and Management*. Bonadonna, G. (ed.) J. Wiley and Sons: Chichester, p. 229.

RODRIGUES, L.M., MIDWOOD, C., COOMBES, R.C., STEVENS, A.N., STUBBS, M. & GRIFFITHS, J.R. (1988). $^{31}P$ Nuclear magnetic resonance spectroscopy studies of the response of rat mammary tumours to endocrine therapy. *Cancer Res.*, **48**, 89.

ROFSTAD, E.K., DEMUTH, P., FENTON, B.M. & SUTHERLAND, R.M. (1988). $^{31}P$ Nuclear magnetic resonance spectroscopy studies of tumours energy metabolism and its relationship to intracapillary oxyhaemoglobin saturation status and tumour hypoxia. *Cancer Res.*, **48**, 5440.

STEEN, R.G. (1989). Response of solid tumours to chemotherapy monitored by *in vivo* $^{31}P$ nuclear magnetic resonance spectroscopy: a review. *Cancer Res.*, **49**, 4075.

STEEN, R.G., TAMARGO, R.J., MCGOVERN, K.A. & 4 others (1988). *In vivo* $^{31}P$ nuclear magnetic resonacne spectroscopy of subcutaneous 9L gliosarcoma: effects of tumour growth and treatment with 1,3,-bis (2-chloroethyl)-1-nitrosourea on tumour bioenergetics and histology. *Cancer Res.*, **48**, 676.

STUBBS, M., COOMBES, R.C., GRIFFITHS, J.R., MAXWELL, R.J., RODRIGUES, L.M. & GUSTERSON, B.A. (1990) $^{31}p$ Spectroscopy and histological studies of the response of rat mammary tumours to endocrine therapy. *Br. J. Cancer*, **61**, 258.

STUBBS, M., RODRIGUES, L.M. & GRIFFITHS, J.R. (1989). Growth studies of subcutaneous rat tumours: comparison of $^{31}P$ NMR spectroscopy, acid extracts and histology. *Br. J. Cancer*, **60**, 71.
TOZER, G.M., BHUJWALLA, Z.M., GRIFFITHS, J.R. & MAXWELL, R.J. (1989). Phosphorus-31 magnetic resonance spectroscopy and blood perfusion of the RIF-1 tumour following X-irradiation. *Int. J. Radiat. Oncol. Biol. Phys.*, **16**, 155.
VAUPEL, P., FORTMEYER, H.P., RUNKEL, S. & KALLINOWSKI, F. (1987). Blood flow, oxygen consumption and tissue oxygenation of human breast cancer xenografts in nude rats. *Cancer Res.*, **47**, 3496.


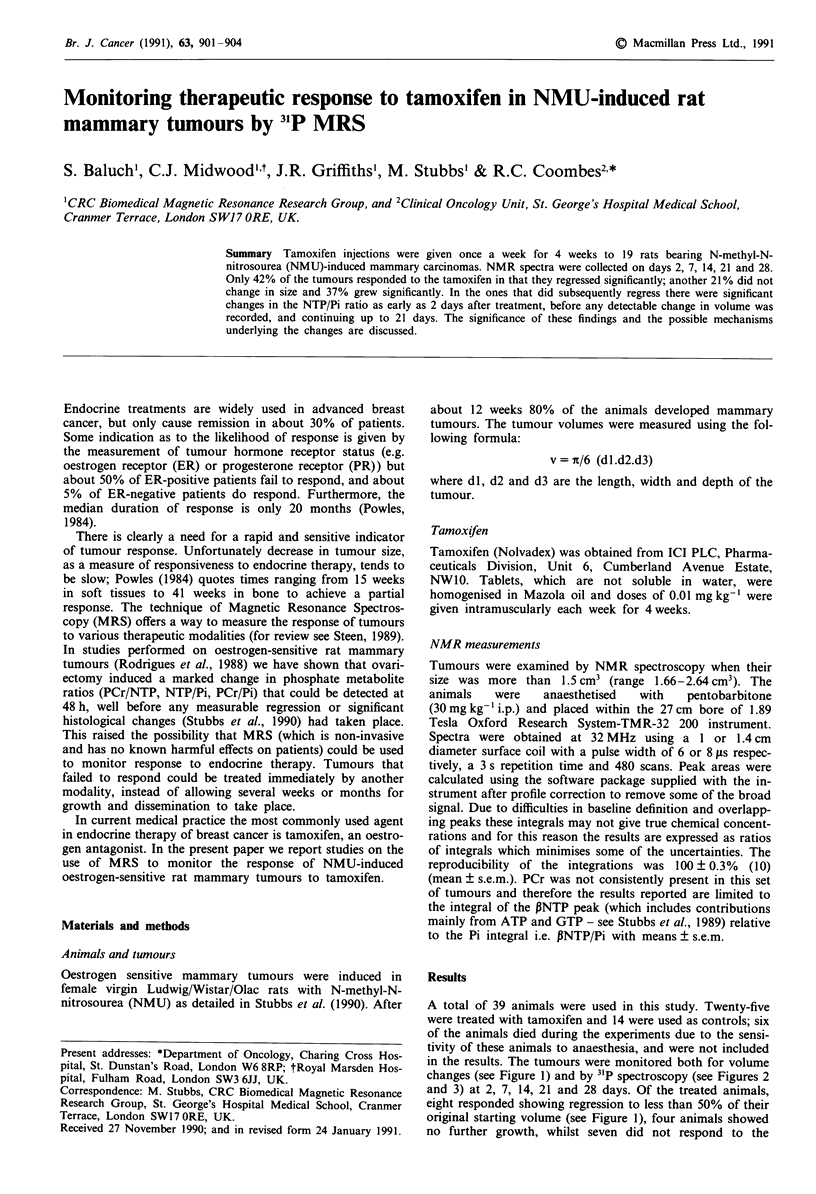

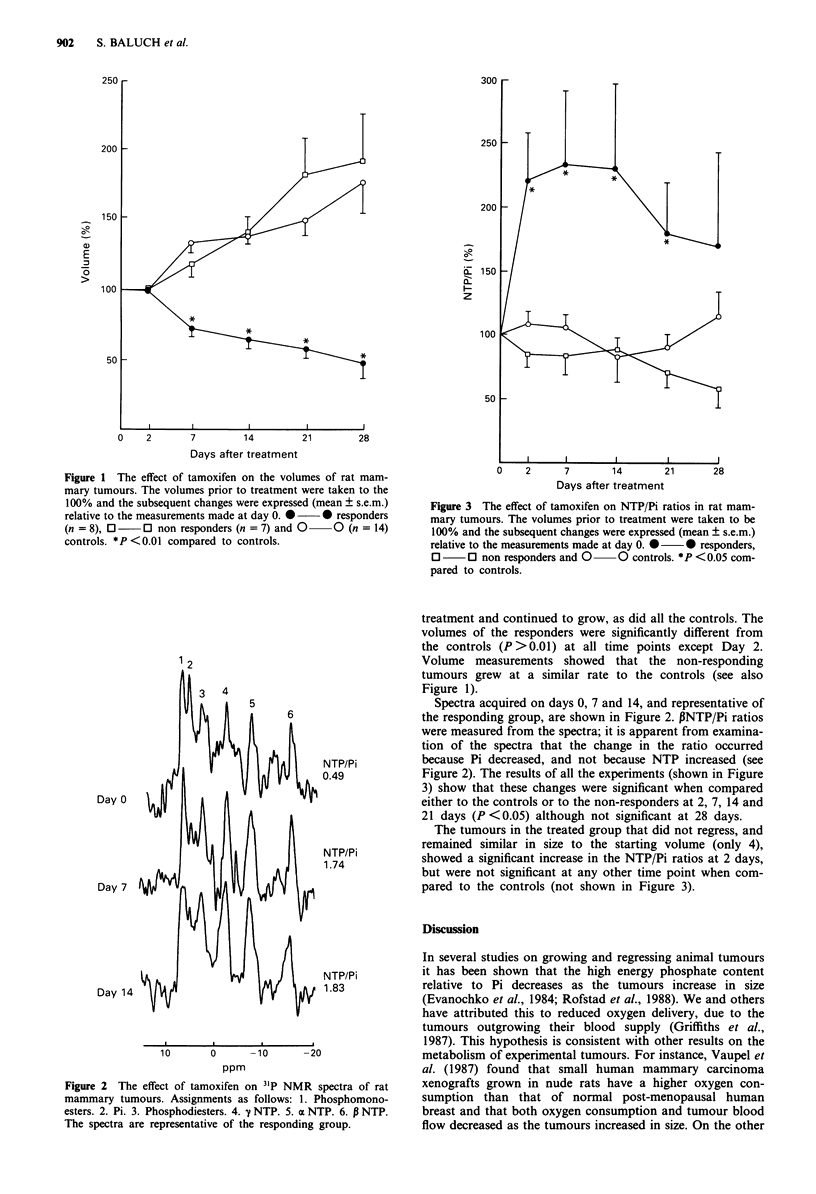

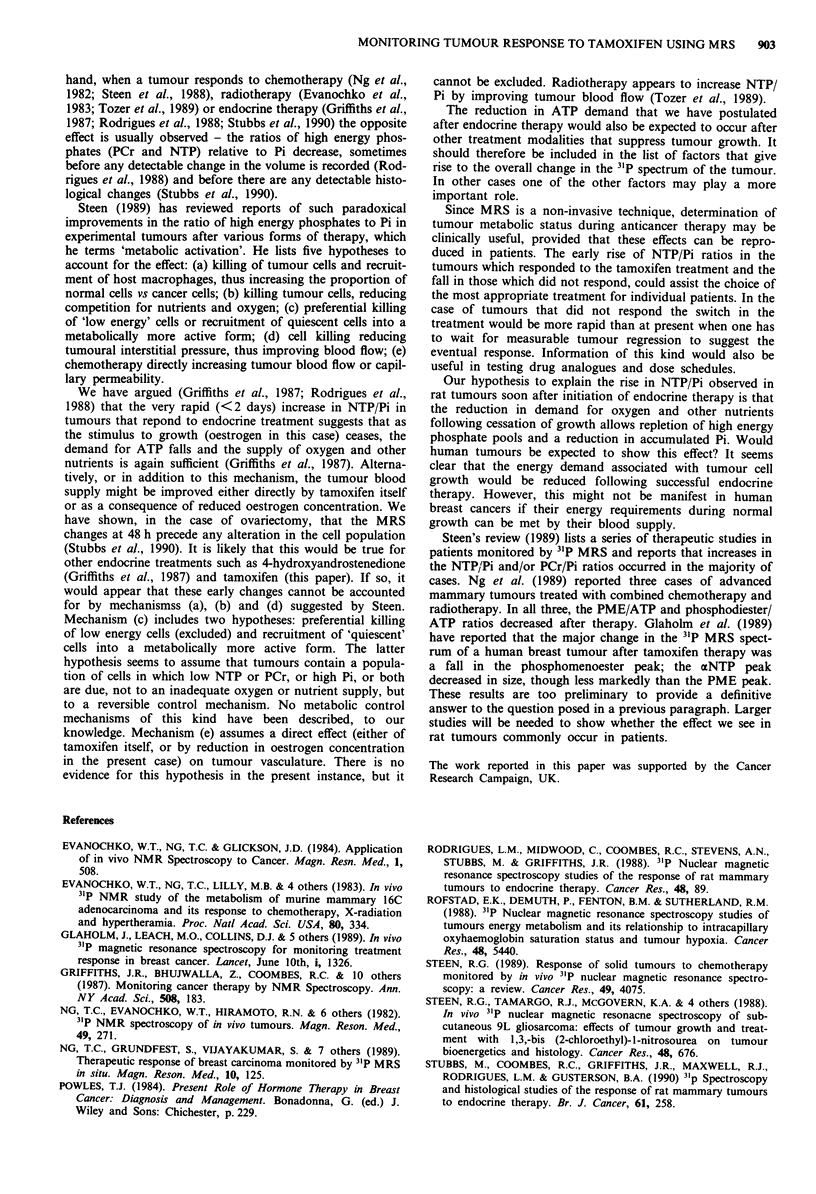

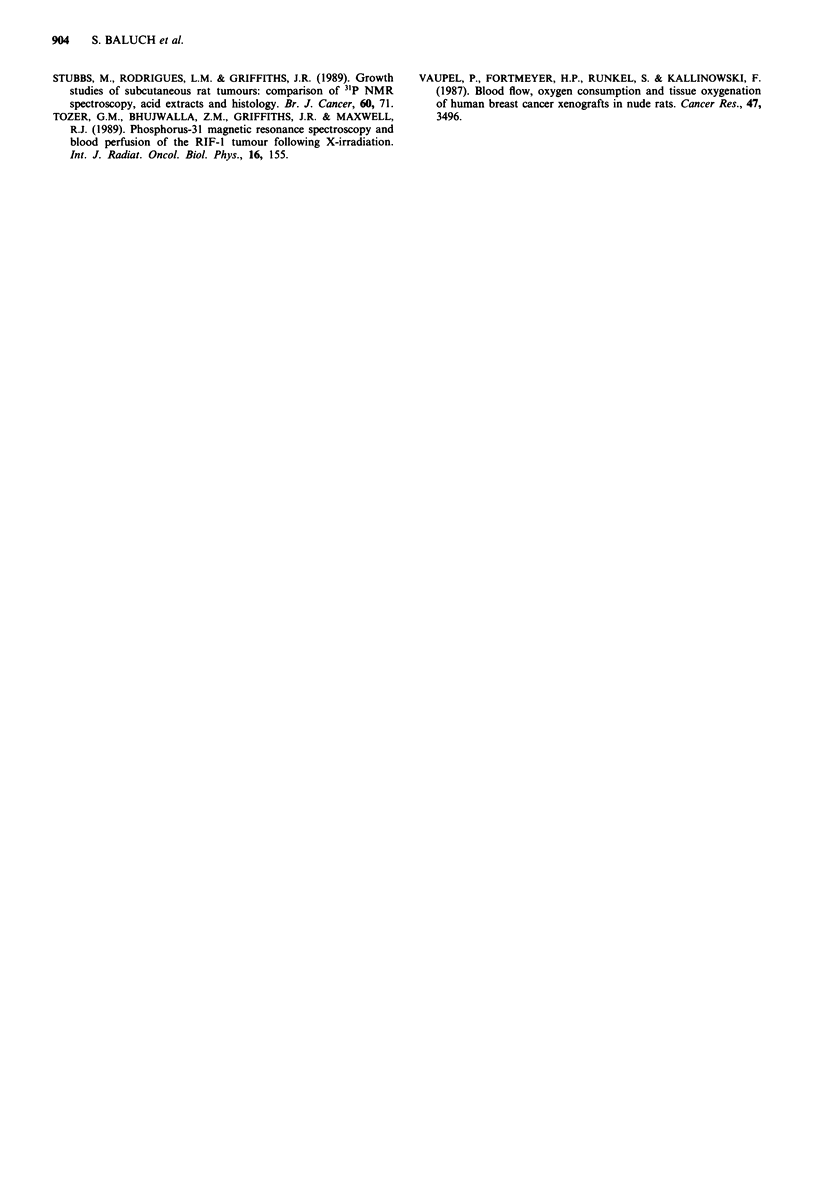


## References

[OCR_00360] Evanochko W. T., Ng T. C., Glickson J. D. (1984). Application of in vivo NMR spectroscopy to cancer.. Magn Reson Med.

[OCR_00365] Evanochko W. T., Ng T. C., Lilly M. B., Lawson A. J., Corbett T. H., Durant J. R., Glickson J. D. (1983). In vivo 31P NMR study of the metabolism of murine mammary 16/C adenocarcinoma and its response to chemotherapy, x-radiation, and hyperthermia.. Proc Natl Acad Sci U S A.

[OCR_00373] Glaholm J., Leach M. O., Collins D. J., Mansi J., Sharp J. C., Madden A., Smith I. E., McCready V. R. (1989). In-vivo 31P magnetic resonance spectroscopy for monitoring treatment response in breast cancer.. Lancet.

[OCR_00378] Griffiths J. R., Bhujwalla Z., Coombes R. C., Maxwell R. J., Midwood C. J., Morgan R. J., Nias A. H., Perry P., Prior M., Prysor-Jones R. A. (1987). Monitoring cancer therapy by NMR spectroscopy.. Ann N Y Acad Sci.

[OCR_00386] Ng T. C., Grundfest S., Vijayakumar S., Baldwin N. J., Majors A. W., Karalis I., Meaney T. F., Shin K. H., Thomas F. J., Tubbs R. (1989). Therapeutic response of breast carcinoma monitored by 31P MRS in situ.. Magn Reson Med.

[OCR_00396] Rodrigues L. M., Midwood C. J., Coombes R. C., Stevens A. N., Stubbs M., Griffiths J. R. (1988). 31P-nuclear magnetic resonance spectroscopy studies of the response of rat mammary tumors to endocrine therapy.. Cancer Res.

[OCR_00402] Rofstad E. K., DeMuth P., Fenton B. M., Sutherland R. M. (1988). 31P nuclear magnetic resonance spectroscopy studies of tumor energy metabolism and its relationship to intracapillary oxyhemoglobin saturation status and tumor hypoxia.. Cancer Res.

[OCR_00409] Steen R. G. (1989). Response of solid tumors to chemotherapy monitored by in vivo 31P nuclear magnetic resonance spectroscopy: a review.. Cancer Res.

[OCR_00414] Steen R. G., Tamargo R. J., McGovern K. A., Rajan S. S., Brem H., Wehrle J. P., Glickson J. D. (1988). In vivo 31P nuclear magnetic resonance spectroscopy of subcutaneous 9L gliosarcoma: effects of tumor growth and treatment with 1,3-bis(2-chloroethyl)-1-nitrosourea on tumor bioenergetics and histology.. Cancer Res.

[OCR_00421] Stubbs M., Coombes R. C., Griffiths J. R., Maxwell R. J., Rodrigues L. M., Gusterson B. A. (1990). 31P-NMR spectroscopy and histological studies of the response of rat mammary tumours to endocrine therapy.. Br J Cancer.

[OCR_00433] Tozer G. M., Bhujwalla Z. M., Griffiths J. R., Maxwell R. J. (1989). Phosphorus-31 magnetic resonance spectroscopy and blood perfusion of the RIF-1 tumor following X-irradiation.. Int J Radiat Oncol Biol Phys.

[OCR_00439] Vaupel P., Fortmeyer H. P., Runkel S., Kallinowski F. (1987). Blood flow, oxygen consumption, and tissue oxygenation of human breast cancer xenografts in nude rats.. Cancer Res.

